# Efficacy of MLN9708 (ixazomib) in experimental autoimmune myasthenia gravis and in anti-AChR producing primary thymic cell cultures from myasthenia gravis patients

**DOI:** 10.3389/fimmu.2025.1521432

**Published:** 2025-05-15

**Authors:** Marina Mané-Damas, Abhishek Saxena, Gisela Nogales-Gadea, Jo Stevens, Shannen Vincken, Maarten van Beek, Nynke J. van den Hoogen, Elbert A. J. Joosten, Nick Willcox, Hans Duimel, Jos G. Maessen, Peter C. Molenaar, Marc H. De Baets, Mario Losen, Pilar Martinez-Martinez

**Affiliations:** ^1^ Department of Psychiatry and Neuropsychology, Mental Health and Neuroscience Research Institute, Faculty of Health, Medicine and Life Sciences, Maastricht University, Maastricht, Netherlands; ^2^ Advanced Biologics Design Group, Center for Translational Research, Shenzhen Bay Laboratory, Shenzhen, China; ^3^ Badalona Neuromuscular Research Group (GRENBA), Institut d'Investigació en Ciències de la Salut Germans Trias i Pujol (IGTP), Universitat Autònoma de Barcelona, Badalona, Spain; ^4^ Department of Anesthesiology, Mental Health and Neuroscience Research Institute, Faculty of Health, Medicine and Life Sciences, Maastricht University, Maastricht, Netherlands; ^5^ Department of Paediatrics, University of Calgary, Calgary, AB, Canada; ^6^ Hotchkiss Brain Institute, University of Calgary, Calgary, AB, Canada; ^7^ Department of Clinical Neurosciences, Weatherall Institute for Molecular Medicine, University of Oxford, Oxford, United Kingdom; ^8^ Microscope CORE lab, Maastricht University, Maastricht, Netherlands; ^9^ Department of Cardiothoracic Surgery, Maastricht UMC+, Maastricht, Netherlands

**Keywords:** ixazomib, targeting autoimmune plasma cells, myasthenia gravis, proteasome inhibition, autoantibody, therapy, autoimmunity

## Abstract

Proteasome inhibitors can eliminate malignant, alloreactive, or autoreactive plasma cells. These cells are key players in antibody-mediated autoimmune disorders and thus suitable therapeutic targets for these drugs. However, certain proteasome inhibitors cause toxic peripheral neuropathy in patients. Ixazomib (MLN9708, Ninlaro), an oral proteasome inhibitor, has a more favorable safety profile in multiple myeloma patients. Here we tested its efficacy in preventing and treating experimental autoimmune myasthenia gravis (EAMG). Female Lewis rats were treated with two subcutaneous doses of 0.35 mg/kg of ixazomib per week, starting either 4 weeks before or at disease onset; both substantially lowered final total IgG and rat acetylcholine receptor (AChR) autoantibody levels. Interestingly, two weekly doses of 0.20 mg/kg of ixazomib for the last 4 weeks did not reduce autoantibody levels. A single dose of 0.50 mg/kg was acutely toxic in rats. In cultures of thymic cells from early-onset myasthenia gravis (EOMG) patients, 30 nM ixazomib or higher almost completely eliminated plasma cells and halted their IgG and AChR antibody production. We conclude that proteasome inhibition with ixazomib effectively depletes plasma cells from MG patients *in vitro* and in a rat model *in vivo*. These results encourage further investigations into therapeutic plasma cell targeting for MG patients.

## Introduction

Plasma cells are terminally differentiated, non-dividing effector cells of the B cell lineage that have lost many surface markers. They are high-rate antibody-secreting cells (>5,000 molecules per sec), and typically reside in lymphoid tissues (1–4). Besides their involvement in several neoplasms, including multiple myeloma (MM), long-lived plasma cells (LLPCs) play an important role in many antibody-mediated autoimmune disorders. They are resistant to radiation, CD19 and CD20 antibody (Ab) therapies, glucocorticoids and other immunosuppressive drugs. This strongly implicates autoreactive LLPCs in the delays in responses or even resistance to such therapies (5–8), that are often observed in some antibody-mediated autoimmune disorders.

Myasthenia gravis (MG) is a classic antibody-mediated autoimmune disorder affecting the neuromuscular junction (NMJ). It is characterized by fatigable skeletal muscle weakness, and about 10% of patients are treatment-resistant. It is caused by autoantibodies against the muscle nicotinic acetylcholine receptor (AChR) in 85% of MG patients (AChR-MG) (9–12). Autoantibodies against proteins involved in the clustering of AChR at the NMJ have also been identified in some MG patients. Around 5% of MG patients without AChR antibodies present antibodies against the muscle specific kinase (MuSK) (13, 14) and only 1-5% against the Low-density lipoprotein receptor-related protein 4 (Lrp4) (15–17). In another ~5%, with “seronegative” MG, targets are yet to be identified.

In the well-defined subgroup of AChR-MG with onset before age 50 (Early-onset MG; EOMG), the thymus is strongly implicated in pathogenesis (18–20), and its removal by thymectomy is clinically beneficial (21). An EOMG thymus typically shows follicular hyperplasia, with autoreactive (and other) T, B, and plasma cells in and near numerous ectopic lymphoid follicles with germinal centers (19, 22–25). These plasma cells have a high capacity to secrete AChR autoantibodies spontaneously, whether cultured directly after surgery or after cryo-preservation (19, 20, 24, 25).

LLPCs are resistant to various conventional therapies, including rituximab (anti-CD20), and can persist in the bone marrow, continuously producing autoantibodies as previously mentioned. In contrast, short-lived plasma cells (SLPCs) have a faster turnover and are more responsive to B cell-targeting therapies like rituximab. Patients with MuSK-MG respond better to rituximab than those with AChR-MG (26). This difference suggests that MuSK antibodies are primarily derived from SLPCs, which are vulnerable to CD20 depletion. Conversely, AChR-MG likely involves a higher proportion of LLPCs, which do not express CD19 or CD20, rendering them resistant to rituximab and other B cell depletion therapies (12).

Other immunosuppressive drugs such as prednisone and azathioprine are generally used for long-term MG therapy (27). Because azathioprine, mycophenolate and methotrexate only act on dividing lymphocytes, they prevent the generation of LLPCs from B cells but do not affect those that are already terminally differentiated and therefore persist in the tissues. Consequently, the autoantibody levels only start to drop after many months (28) or even years (29), depending on the lifespan/turnover of the plasma cells, which therefore demand interim direct targeting to ‘buy time’ while the immunosuppressants are taking effect. Their high rate of immunoglobulin (Ig) (30) synthesis renders LLPCs particularly sensitive to inhibition of the proteasome, leading to build-up of misfolded proteins within the endoplasmic reticulum (ER), activation of the terminal unfolded protein response and apoptosis (31–33).

Bortezomib (Velcade) is a first-in-class proteasome inhibitor, which binds reversibly to the 26S proteasome and is currently approved for the treatment of multiple myeloma (MM) and mantle cell lymphoma (34). In addition, it has been used for treatment-refractory NMDA receptor autoimmune encephalitis, alone or in combination with other immunosuppressive agents, as well as for persistent childhood-onset neuropsychiatric systemic lupus erythematosus, leading to clinical improvement or remission (35–37). Previously, we examined the effect of bortezomib in EOMG thymic cell cultures, showing efficient depletion of LLPCs and reduction of IgG and autoantibody production (38); also in the rat experimental autoimmune MG model (EAMG), where it led both to significant clinical improvements and to a reduction of post-synaptic damage (39). Additionally, it was also shown to have a promising potential for the treatment of systemic lupus erythematosus (SLE) in a preclinical model (40).

A novel proteasome inhibitor, ixazomib (MLN9708 or its active form MLN2238), is currently indicated for the treatment of MM patients who had at least 1 prior therapy, and is under clinical investigation for other malignancies and plasma cell disorders and for multiple sclerosis. It is an N-capped dipeptidyl leucine boronic acid, which inhibits the proteolytic activity of the 20S unit of the proteasome and is even effective for tumors that are bortezomib-resistant in preclinical models (41). Importantly, clinical trials have demonstrated that ixazomib shows a significantly lower risk of neurotoxicity (42); particularly in the treatment of MM, there were consistently fewer cases of less severe peripheral neuropathy with ixazomib than bortezomib (43, 44), which is attributed to its more favorable pharmacokinetic profile, shorter half-life and reduced drug exposure to peripheral nerves (45).

These observations, therefore, encouraged us to investigate ixazomib (a) on IgG and AChR antibody production by EOMG thymic cells *in vitro*; and (b) in the active immunization rat model of MG.

## Patients, materials and methods

### Patients

**Figure 1:**
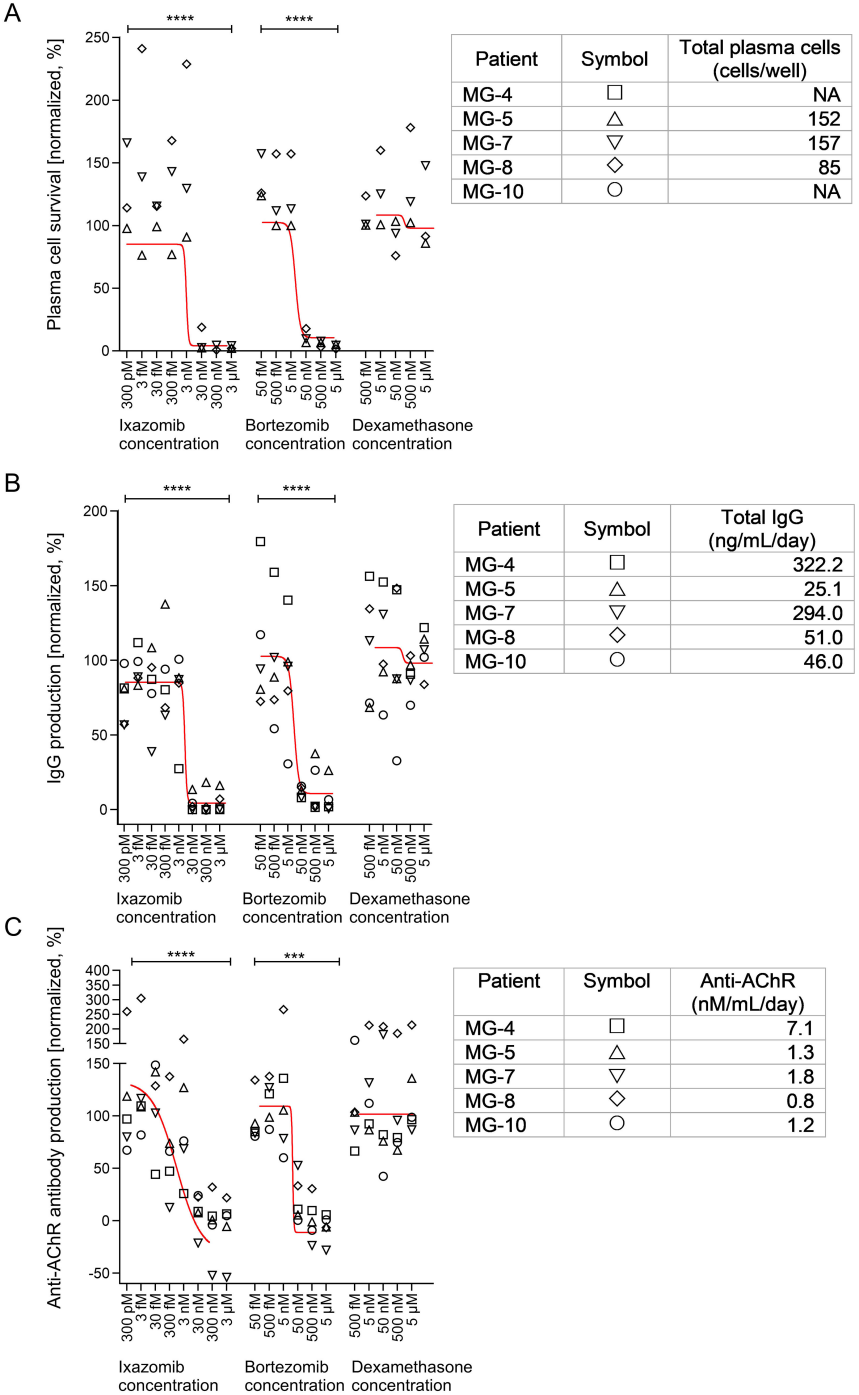
Effects of ixazomib, bortezomib and dexamethasone on plasma cell numbers and function in EOMG thymic cell cultures. **(A)** Normalized number of surviving plasma cells on day 15. **(B)** Production of total IgG and **(C)** human AChR autoantibodies between days 12 and 15. Sigmoidal tendency lines using the average of each treatment dose and drug are shown in red. Each point represents an average of 3 individual wells (per condition) from the indicated patients. In each graph the results were expressed as a percentage of the corresponding control cultures without experimental drugs. The absolute values are given in the tables on the right for each patient and represent the untreated conditions for each parameter. One-way ANOVA and Bonferroni *post hoc* testing were used for statistical analyses. ***p <0.001 and ****p<0.0001. NA, not available/tested.

Thymus samples were taken at routine thymectomy with informed consent and Ethics Committee approval, dispersed and cryopreserved as described (25). The five EOMG donors had not been pre-treated with glucocorticoids and had high serum levels of anti-AChR antibodies (71 to >500 nM), which their thymic cells were known to produce in culture [**Figure 1** (38)]; their details are summarized in **Supplementary Table S1**.

### EOMG thymic cell culture and drug treatment

Thymic cells from EOMG patients were dispersed with dispase and collagenase and cryopreserved in liquid nitrogen as reported (19, 25). Briefly, thymic cells were pre-cultured for 3-7 days before adding the drugs to allow recovery from the thawing procedure, adaptation to culture conditions, and for measuring antibody production at baseline (38). Bortezomib (Btz; Velcade, Janssen-Cilag B.V., Belgium) and ixazomib (Ixa; Takeda Development Center Americas, Inc., Lexington MA) were dissolved in sterile saline, and dexamethasone (Dex; D4902; Sigma Aldrich) in absolute ethanol. Drugs were added – or not – on days 8 and 12 at the indicated concentrations (**Figure 1**). Surviving plasma cells were enumerated on day 15 by immunofluorescence as specified below. Total and viable cells were also quantified using the TC20 automatic cell counter (Biorad) on day 15 (38).

### Immunofluorescence staining and enumeration of plasma cells by microscopy

Plasma cells on each glass slide were identified by their intracellular IgG staining, eccentric ‘cartwheel’ nuclei, their distinct shape and size. They were counted by two blinded researchers on an Olympus BX51 fluorescence microscope as described (38). Their absolute numbers were determined and normalized to those in cultures without added drugs.

### EAMG rat model

Female Lewis rats were purchased from Charles River. The experiments were approved by the Committee on Animal Welfare (Project License number 2013-095) in accordance with European and Dutch laws, rules, and guidelines (86/609/EU). 6 week-old rats were weighed and allocated to a dose-finding study (n=72) or a treatment-timing study (n=84).

EAMG was induced by active immunization with *Torpedo* AChR (tAChR) following standard guidelines (46), except that the dose was 20 μg tAChR per animal (39). Briefly, 7 week-old rats were injected subcutaneously (sc) around the base of the tail (dorsally, at the level of the sacrum) with 200 μL emulsion of tAChR in complete Freund’s adjuvant (CFA). Control animals (non-EAMG) were immunized with an equivalent volume of emulsified saline/CFA. Animals were euthanized 8 weeks post-immunization, or earlier if they reached humane endpoints.

### Experimental design and drug administration

Lyophilized ixazomib and bortezomib (Takeda Development Center Americas, Inc.) were dissolved in sterile saline. In the initial dose-finding study, 4 week-immunized and control rats received bi-weekly sc 500 µL injections (4-8w) in the neck containing ixazomib solution (0.20 mg/kg, 0.35 mg/kg or 0.50 mg/kg) or saline (**Supplementary Table S2**). In the follow-up treatment-timing study, animals were injected sc either (i) twice weekly directly after immunization with either saline or 0.35 mg/kg ixazomib, for 8 weeks as a preventive strategy (0-8w), or (ii) with saline for the first 4 weeks and with 0.35 mg/kg ixazomib or 0.20 mg/kg bortezomib for the following 4 weeks as delayed treatment (4-8w) (**Supplementary Table S2**). For simplicity, the results of these two studies are described together.

### Clinical scoring

Animals were observed daily and weighed weekly both as an index of general health and for dose-evaluation, and tested for muscle weakness and fatigue as described (39, 46). Briefly, all animals were also tested weekly (while held manually near the base of the tail) for their ability to: 1) grasp and lift a 300 g metal grid for 30 sec repeatedly; 2) climb once to the back of the hand. Clinical scoring was performed weekly by a blinded researcher as described (39): “0” = no abnormalities observed; “1” = weakness evident only after exercise (i.e. reduced activity when returning the animal to the cage after testing); “2” = clinical signs evident before/during exercise (i.e. incapacity to finish the test); “3” = severe weakness/illness before testing (i.e. no grasp or moribund). Humane endpoints were defined as a clinical score of 3 and/or >20% weight loss; any such animal was euthanized within 24 h.

### Analysis of mechanical and thermal (pain) sensitivity

The Von Frey and Hargreaves tests of withdrawal thresholds to mechanical or thermal paw stimulation were used as described (47, 48) to assess any effects of possible sensory neuropathy induced by the experimental drugs. Mechanical sensitivity was assessed by plantar application of calibrated Von Frey filaments to the left hind-paw, logarithmically increasing in force (1.202; 2.041; 3.63; 5.495; 8.511; 15.136; and 28.84 g) using the up-down method described by Chaplan (47). Thermal sensitivity was determined using a focus radiant heat source (Plantar test apparatus 37370, Ugo-Basille, Italy) under the left hind-paw until the paw was withdrawn. A cut-off score of 20.1 sec was used to prevent tissue damage. For both tests, only withdrawal responses associated with aversive behavior to the stimulus (e.g. paw licking, postural changes, and/or attacking of the filament at paw stimulation) were scored. For the Von Frey test, the registered 50% paw withdrawal threshold (WT) measured in grams was logarithmically transformed to obtain a linear scale. Tests were performed on alternate weeks by a blinded researcher.

### Sensory nerve conduction velocity measurements

Before starting the treatment and every second week thereafter, a blinded researcher determined the sensory nerve conduction velocity (SNCV) in the tail of each animal as described (49). Briefly, the antidromic SNCV in the tail nerve was assessed by placing recording ring electrodes distally in the tail, while the stimulating ring electrodes were placed 5 cm and 10 cm proximally to the recording point. The latencies of the potentials recorded at the 2 sites after nerve stimulation were determined (peak-to-peak) and nerve conduction velocity was calculated accordingly.

### Rat bone marrow plasma cell quantification by electron microscopy

For electron microscopy (EM) analysis, bone marrow was flushed out of rat femora in 1 mL of 3% glutaraldehyde in phosphate buffer and processed as described (39). Plasma cells were identified by their “cartwheel” chromatin configuration in the nucleus, and extensive rough ER in the cytoplasm, indicative of high protein synthetic capacity. To guard against any differential sedimentation, they were counted in sections taken at six different levels in the pellets. For each sample, ~800 bone marrow cells were counted and the results were expressed in percentages as survival rate of plasma cells.

### Measurement of total human and rat IgG and anti-rat AChR autoantibody levels

Total human IgG levels in thymic cell cultures and rat plasma IgG levels were measured by a standard sandwich ELISA as described (38, 39). The concentration of anti-rat AChR autoantibodies was measured by radioimmunoprecipitation assay (RIA) as reported (38, 39).

## Results

### Depletion of plasma cells in primary thymus cell cultures

We determined concentrations of ixazomib and bortezomib required to deplete plasma cells in each EOMG patient’s thymus cell cultures (**Figure 1A**). At ≥30 nM ixazomib, very few plasma cells were detected, but their numbers were unaffected at ≤3 nM [p<0.001]. In parallel, depletions by bortezomib were similar at ≥50 nM but not evident at ≤5 nM [p<0.01]. We next assessed general cell toxicity of these drugs. As *in vivo*, many thymic cells normally die in culture (19). Unsurprisingly, by day 15, we observed 57% loss of total cells in control cultures. Relative survival still averaged ~70% of controls at 30 nM ixazomib and 50 nM bortezomib, levels that reduced plasma cells by almost 100% (**Supplementary Figure S1**). Only at higher concentrations were total cell numbers substantially decreased. In view of the reported niche-dependence of plasma cells, their long survival in our standard untreated cultures is remarkable, though long known (19, 24, 25).

Dexamethasone had no significant effects over the range of 0.1 - 1000 nM (**Figure 1A**), at which it is known to inhibit human lymphocyte proliferation (50, 51). Nor did it affect plasma cell activity (**Figures 1B, C**) or survival in these cultures (**Figure 1**, **Supplementary Figure S1**).

Additionally, we analyzed secretion rates of total IgG and AChR autoantibodies into culture supernatants. As expected, their dose-sensitivities to ixazomib and bortezomib were very similar to those observed above for plasma cell survival for each donor (**Figures 1B, C**). By contrast, no significant differences were noticed across the dexamethasone dose-range. This further confirms that human (autoreactive) plasma cells are susceptible to proteasome inhibitors and are the main spontaneous AChR autoantibody-producing cell type in these cultures (14, 24, 38).

### Ixazomib prevents weight loss in EAMG animals

We assessed the safety and efficacy of the drug in the EAMG rat model in a first dose-finding study. Doses of 0.20 and 0.35 mg/kg ixazomib were well tolerated by all animals. At 0.50 mg/kg it proved toxic: 8 out of 9 animals died or had to be euthanized within the first 24 hours (**Supplementary Figure S2**). In a follow-up treatment-timing study, 0.35 mg/kg ixazomib was used to optimize treatment regimens including: (i) a prevention arm, starting drug at the time of immunization, 4 weeks before EAMG onset, continuing until the endpoint after a further 4 weeks [0-8w Ixa 0.35 mg/kg] and (ii) a delayed treatment arm, where the drug was injected only for the last 4 weeks, from the usual time of EAMG onset [4-8w Ixa 0.35 mg/kg].

**Figure 2:**
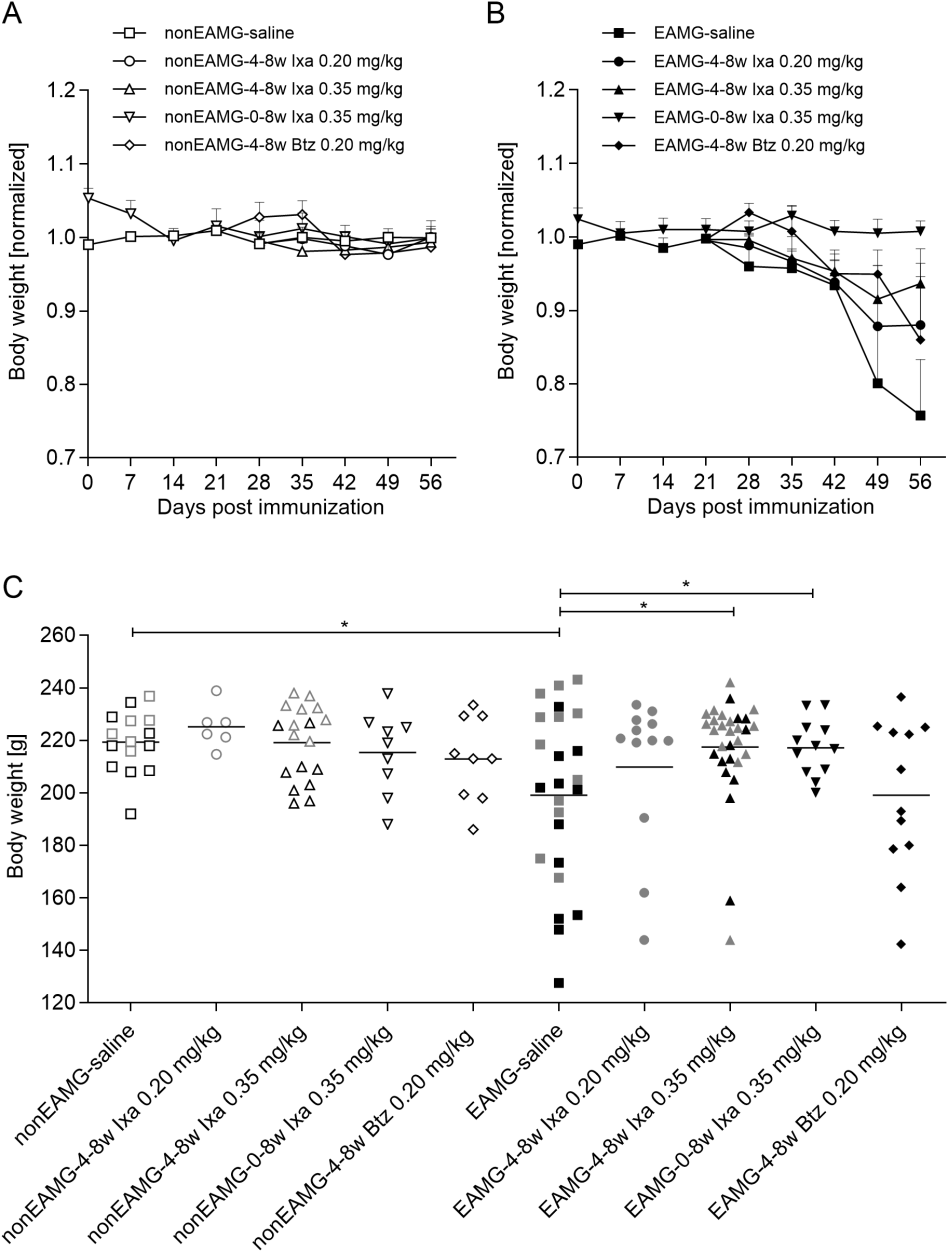
Rat body weights: combined for all animals tested in the dose-finding and treatment-timing studies. Weekly body weights in **(A)** non-EAMG rats and **(B)** EAMG rats, averaged for each group and shown as ratios to the averages of the non-EAMG-saline treated controls. **(C)** Individual body weights at the end of the experiment [after 8 weeks or earlier if animals reached the humane endpoint]. Open and filled symbols show the non-EAMG groups and EAMG groups respectively. Grey and black symbols represent rats in the dose-finding and treatment-timing studies respectively. One-way ANOVA, multiple comparison of the indicated groups with Bonferroni *post hoc* testing were used for statistical analyses. *p<0.05.

During the first 4 weeks, all animals gained weight gradually, a trend that continued for all non-EAMG control animals, regardless of drug dosage (**Figure 2A**). After 4 weeks, all EAMG animals lost at least some weight, except for those given 0.35 mg/kg ixazomib (**Figures 2B, C**); that was more pronounced after treatment with saline or the low dose of ixazomib (**Figure 2B**). By the end of the experiment, weight loss was significantly less than in the controls after treatment with 0.35 mg/kg ixazomib – whether for only the last 4 weeks or especially for the full time-course [p<0.05] - whereas there was no significant protection with 0.20 mg/kg of either drug (**Figure 2C**).

At most, one rat in each group for each experiment had to be euthanized prematurely because of severe MG symptoms and resulting weight loss (**Supplementary Figure S2**). The proportion of severely weak rats was slightly lower after 0.35 mg/kg ixazomib for the last 4 weeks, but not significantly so (**Supplementary Figures S3A, B**).

### Ixazomib reduces disease-specific autoantibodies and total IgG

**Figure 3:**
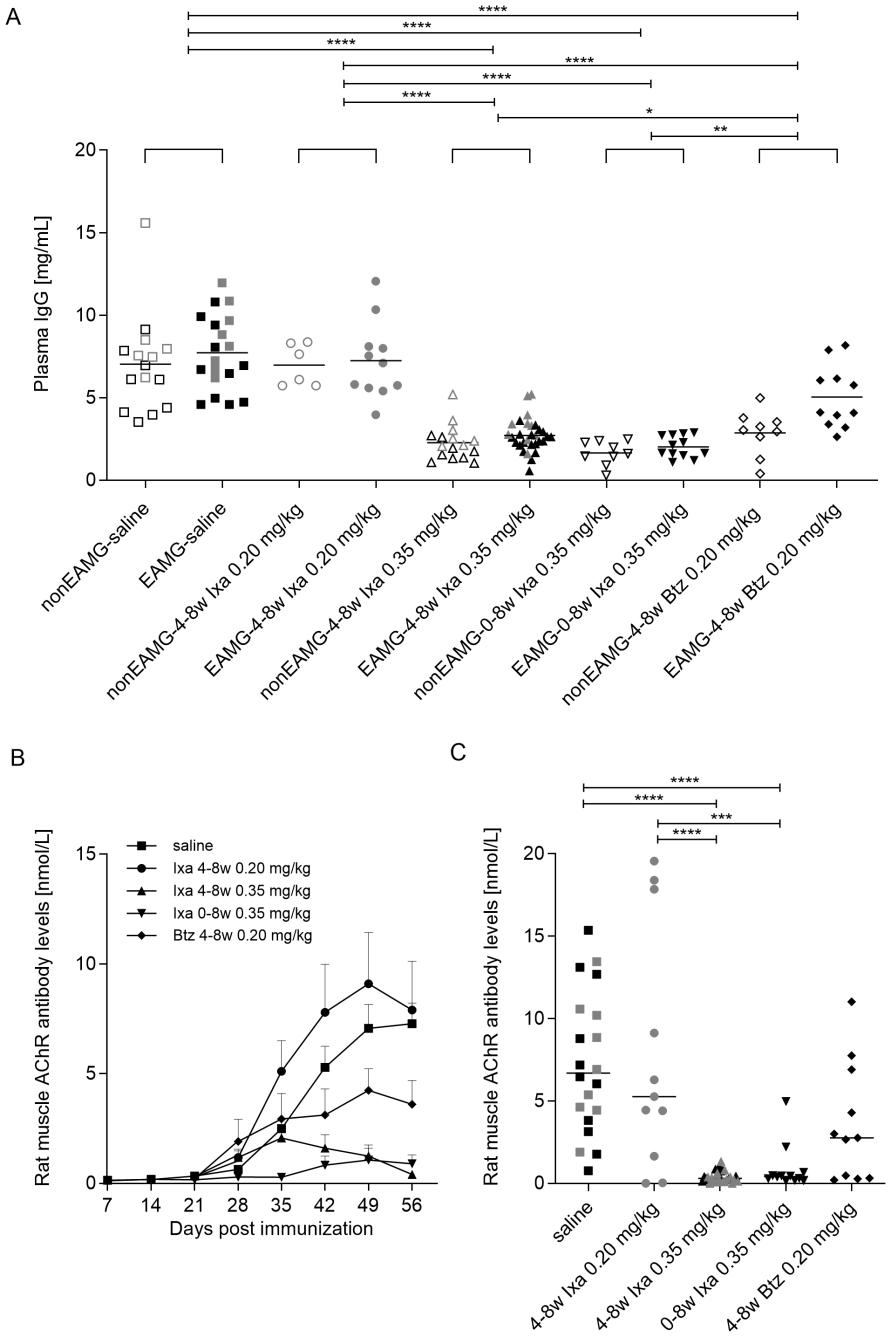
Total IgG and (rat muscle) AChR autoantibody levels. Data were combined to include all animals in the dose-finding and treatment-timing studies. **(A)** Individual plasma IgG levels 8 weeks after immunization. Open and filled symbols show the non-EAMG groups and EAMG groups respectively. **(B)** Group-averaged rat muscle anti-AChR antibody levels over time and at **(C)** 8 weeks post-immunization. Grey and black symbols represent rats in the dose-finding study and treatment-timing studies respectively. One-way ANOVA, multiple comparisons of the indicated columns and Bonferroni *post hoc* testing were used for statistical analyses. *p<0.05, **p< 0.01; ***p <0.001 and ****p<0.0001.

To establish the effects of treatment with ixazomib, we measured IgG levels in the plasma. As expected, they were generally lower and less variable after treatment with 0.35 mg/kg ixazomib or bortezomib than with saline or 0.20 mg/kg ixazomib, both in the non-EAMG and EAMG animals (**Figure 3A**), in agreement with earlier results (39). Administration of 0.35 mg/kg ixazomib for 4 or 8 weeks reduced total plasma IgG significantly more than did 4 weeks of bortezomib (65.5, 75.0 and 45.2% reduction from saline-treated levels respectively 8 weeks post-immunization).

In this model, anti-AChR levels are much higher against *Torpedo* AChR than rat AChR, but only these latter autoantibodies are pathogenic (46); they were starting to rise 4 weeks after immunization in all the present EAMG groups (day 28, **Figure 3B**). Further increases in anti-AChR levels were almost completely prevented until day 56 by treatment with 0.35 mg/kg ixazomib for 8 weeks, and very significantly reduced even in the delayed treatment group (mixed effect analysis with Bonferroni correction for multiple analysis, 28 vs 56 day, p<0.0001). The increase was also largely prevented by 0.20 mg/kg bortezomib compared to saline- and 0.20 mg/kg ixazomib-treatment, where the antibody levels continued increasing. In the latter, the apparent increase was largely due to 3 rats with very high autoantibody levels (**Figure 3C**).

At day 56, reductions in AChR autoantibody levels were more significant and consistent with 0.35 mg/kg ixazomib (by ~ 90% with either regimen) than 0.20 mg/kg bortezomib (reduction of 50.5%, unpaired t test; p=0.0217; **Figure 3C**).

### Ixazomib depletes bone marrow plasma cells in EAMG rats

**Figure 4:**
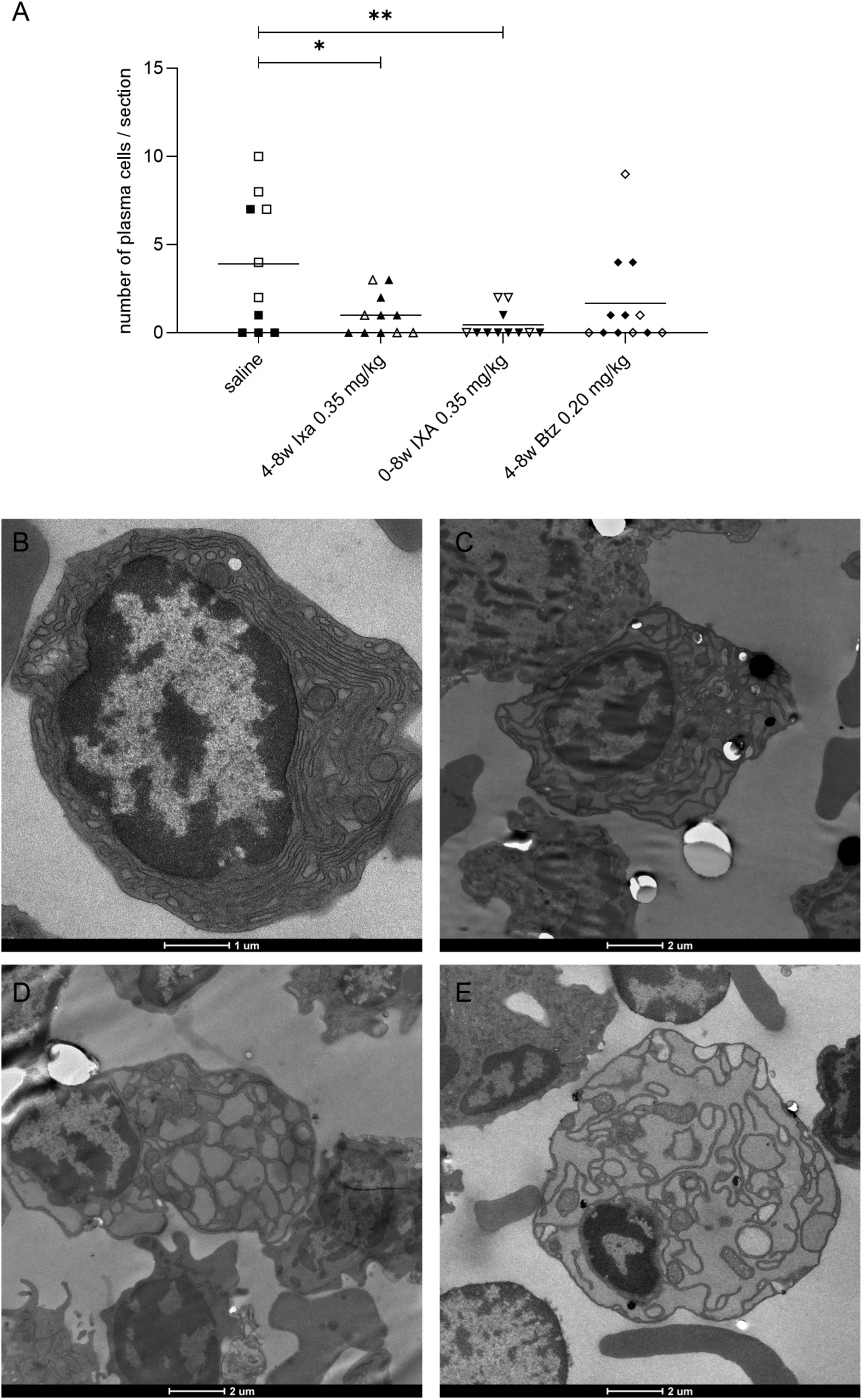
Plasma cells found in bone marrow samples by electron microscopy. **(A)** Numbers of morphologically normal plasma cells. Values for EAMG and non-EAMG rats (filled and empty symbols respectively) were pooled for this analysis. Each point represents a sample from one animal, each with roughly equal total cell numbers; bars = means. Open and filled symbols show the non-EAMG groups and EAMG groups respectively. **(B-E)** Plasma cells were identified by their extensive rough ER, absence of granules, and their cart-wheel-shaped nuclear heterochromatin. The treatments were: **(B)** saline, **(C)** 4-8w Ixa 0.35 mg/kg, **(D)** 0-8w Ixa 0.35 mg/kg and **(E)** 4-8w Btz 0.20 mg/kg. One-way ANOVA, multiple comparison of saline to all treatment groups and Bonferroni *post hoc* testing were used for statistical analyses. *p<0.05; **p<0.01. Scale bar = 1 or 2 µm.

Morphologically normal plasma cells were found by electron microscopy in the bone marrow of all rats tested. Their numbers were significantly reduced in both ixazomib regimens compared to saline-treated animals [p<0.05, One-way ANOVA, Bonferroni *post hoc* testing], though not on bortezomib treatment, because of three outlier animals (**Figure 4A**). We observed some plasma cells with characteristic signs of ER stress in samples from ixazomib- and bortezomib-treated animals (**Figures 4C–E**); and significantly more than in the controls (p=0.02 using the Fisher’s exact test, saline vs proteasome inhibitor treated), despite the low numbers (Saline: 0 out of 39, Ixa 0-8w: 2 out of 8, Ixa 4-8w: 3 out of 14, Btz 4-8w: 3 out of 23).

### Pain sensitivity and nerve conduction

We observed no significant differences in mechanical or thermal pain-sensitivity between the drug- and saline-treated groups (**Supplementary Figure S4**). Nerve conduction velocities measured in the tail significantly increased over time [p<0.0001], but no significant effects of the experimental drugs were observed (**Supplementary Figure S5**), when a mixed-effects analysis using a two-way ANOVA with multiple comparisons and Bonferroni correction was performed.

## Discussion

Our *in vitro* human and *in vivo* rodent studies clearly show that ixazomib strongly reduces plasma cell numbers and activity, almost completely inhibiting the production of both total IgG and AChR autoantibodies in immunized rats.

In our cultures, EOMG thymic plasma cells and their activity were almost equally ablated with appropriate doses of ixazomib and bortezomib. By contrast, they were almost unaffected even by high doses of dexamethasone. That is no surprise (52), as glucocorticoids are well known only to reduce anti-AChR titers and improve muscle strength after 2-3 months in most MG patients; evidently they must do so via mechanisms other than proteasome inhibition.

These rapid and efficient depletions of plasma cells argue strongly that a short initial course of ixazomib might valuably “buy time” while other immuno-suppressive drugs are starting to act. This study thus provides a promising basis for such a clinical trial in AChR-EOMG patients, monitored with MGFA and ADL scores as well as AChR autoantibody levels and single-fiber electromyography. Notwithstanding the narrow “therapeutic window” of ixazomib in rats, it appears to have a favorable safety profile in human clinical studies, even in the few patients available for a trial in lupus nephritis (53).

In the EAMG rat model, subcutaneous doses of 0.35 mg/kg ixazomib twice per week, for 4 or 8 weeks, led to reductions of total serum IgG levels by >65% and of AChR autoantibodies by >88%. They also depleted plasma cells, in line with our present and previous findings with bortezomib (39); the even greater effects of ixazomib probably reflect its selective inhibition of the 20S proteasome and its better tissue penetration than bortezomib (45). Similar reductions of >73% in dsDNA autoantibodies have been observed with ixazomib in a mouse model of SLE (54).

Interestingly, in the delayed treatment arm, ixazomib was no less effective than when continued for the whole 8 weeks. This suggests that proteasome inhibitors affect the late effector phase of the autoimmune response, as we have already seen with bortezomib, where again no differences in plasma cell depletion and rat AChR autoantibody levels were found between the two regimens (39).

Although the trends hint at protection from weakness with either ixazomib or bortezomib treatments, neither achieved significance overall (**Supplementary Figure S3**). In our previous study, treatment with bortezomib did not cure ongoing EAMG, but prevented disease when initiated at the time of immunization (39), in line with the slow recovery of the neuromuscular junction after antibody-induced damage (55, 56). Specifically, its full recovery can take up to 72 days in rodents (57), which parallels clinical observations in humans, where patients may also experience prolonged recovery times (58). Studies suggest that, while initial improvements may be rapid, achieving complete restoration of neuromuscular transmission often requires weeks to months and exceeds the time window of our model.

Furthermore, the failure of clinical benefits to achieve significance could be a result of the lower disease incidence observed in our EAMG model. We chose to immunize with 20 µg tAChR for comparability with our previous study on bortezomib (39), which was conducted before this model was standardized, recommending a dose of 40 µg tAChR (46). Using 40 ug might result in a more robust phenotype. Nevertheless, as shown in the standardized guidelines, the model presents some variability with both doses, clearly exemplified in the levels of autoantibodies found in animals at different timepoints. Readout parameters such as the decrement-inducing curare dose allow us to have a very robust measurement of the AChR loss in the muscle, which is directly associated with the severity of the disease, even in the absence of symptoms (subclinical MG).

The “therapeutic window” of ixazomib was wide in our EOMG-derived thymic cell cultures; we observed significant general cell cytotoxicity only at 30 µM ixazomib. In stark contrast, in the rat EAMG model, the therapeutic window of ixazomib was very narrow: 0.20 mg/kg had no substantive effects, 0.35 mg/kg was well tolerated and immunosuppressive, with no evident adverse effects on peripheral nerve function, while 0.50 mg/kg was not well tolerated in rodents, apparently equivalent to an LD90. This apparent “all or none” response or “quantal dose-response/binary effect”, was evident with only a modest increment in dose of ixazomib. This kind of pharmacodynamics is not unusual, as it has been observed with other high-dose chemotherapy agents such as cisplatin and cyclophosphamide (59).

As compared to bortezomib, ixazomib presents improved pharmacokinetic and pharmacodynamic profiles with a faster proteasome dissociation half-life that probably contributes to its reduced toxicity (45, 60). The lower incidence of ixazomib-related polyneuropathy could be attributed to its high specificity for one of the sites of the 20S proteasome (45) and to lack of the off-target effects observed with bortezomib (61). Thus, ixazomib is preferred in many cases for patients who are at higher risk of developing neuropathy from bortezomib-based regimens (62).

The understanding of neuropathic pain induced by bortezomib and ixazomib is primarily based on data from human patients treated with these drugs, which also served as the rationale for testing ixazomib’s efficacy in MG. However, the EAMG model is not well-suited for assessing neurotoxicity, and this was not the focus of our study. Investigating potential neurotoxicity of bortezomib and ixazomib in the EAMG model was further complicated by the effects of CFA in our Lewis rats, as their sensitivity to mechanical stimuli unexpectedly increased over time, whereas in normal rats, sensitivity reportedly decreases (63).

In conclusion, our present evidence of efficacy argues strongly for a carefully monitored trial of a short early course of ixazomib to “buy time” in EOMG patients while long-term immunosuppressive drugs are starting to take effect.

## Data Availability

The raw data supporting the conclusions of this article will be made available by the authors, without undue reservation.
