# Supplementary material for: Efficacy of MLN9708 (ixazomib) in experimental autoimmune myasthenia gravis and in anti-AChR producing primary thymic cell cultures from myasthenia gravis patients
Source: Front Immunol. 2025 May 15;16:1521432. doi: 10.3389/fimmu.2025.1521432 (PMC12120636; doi:10.3389/fimmu.2025.1521432)
Supplement: Supplementary file 1 [file DataSheet1.docx]

***Supplementary data***

**Table 1.** EOMG patient information.

| Patient | Gender | MG grade | MG onset-age (years) | MG duration at thymectomy (years) | AChR autoantibodies (nM) |
| --- | --- | --- | --- | --- | --- |
| MG-4 | F | 2B | 39 | 1.8 | 71 |
| MG-5 | F | 2A | 30 | 1.9 | 156 |
| MG-7 | F | 2A | 34 | 0.5 | 79 |
| MG-8 | F | 2A | 25 | 13.8 | 500 |
| MG-10 | F | 2A | 20 | 0.7 | >500 |

**Table 2.** Numbers of rats in dose-finding and treatment-timing studies groups.

| Study | Immunization | Saline | 4-8w-Ixa 0.20 mg/kg | 4-8w-Ixa 0.35 mg/kg | 4-8w-Ixa 0.5 mg/kg | 0-8w- Ixa 0.35 mg/kg | 4-8w- Btz 0.20 mg/kg |
| --- | --- | --- | --- | --- | --- | --- | --- |
| Dose-finding study | Control (saline:CFA) | 6 | 6 | 9 | 3 | - | - |
|  | EAMG (tAChR:CFA) | 12 | 12 | 18 | 6 | - | - |
| Treatment-timing study | Control (saline:CFA) | 9 | - | 9 | - | 9 | 9 |
|  | EAMG (tAChR:CFA) | 12 | - | 12 | - | 12 | 12 |

**
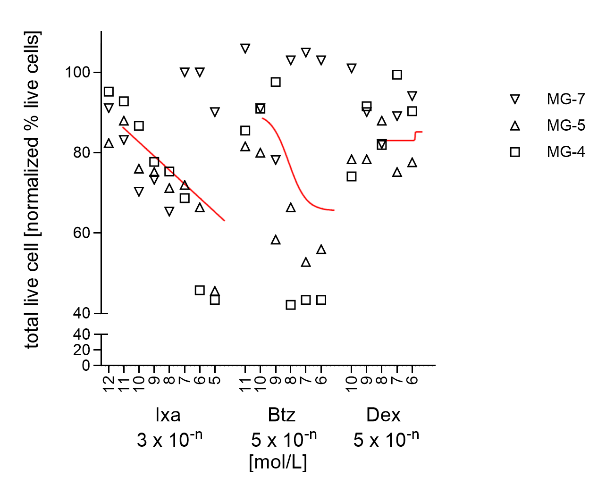
**

**Figure 1.** Effects of ixazomib, bortezomib and dexamethasone on cell viability in EOMG thymic cell cultures from 3 patients (3 replicates/patient/condition). Normalized survival % (each point represents an average of 3 measurements to controls). Tendency lines using the average of each treatment dose and drug are shown in red. One-way ANOVA with multiple comparison and Bonferroni post hoc testing were used for statistical analyses.


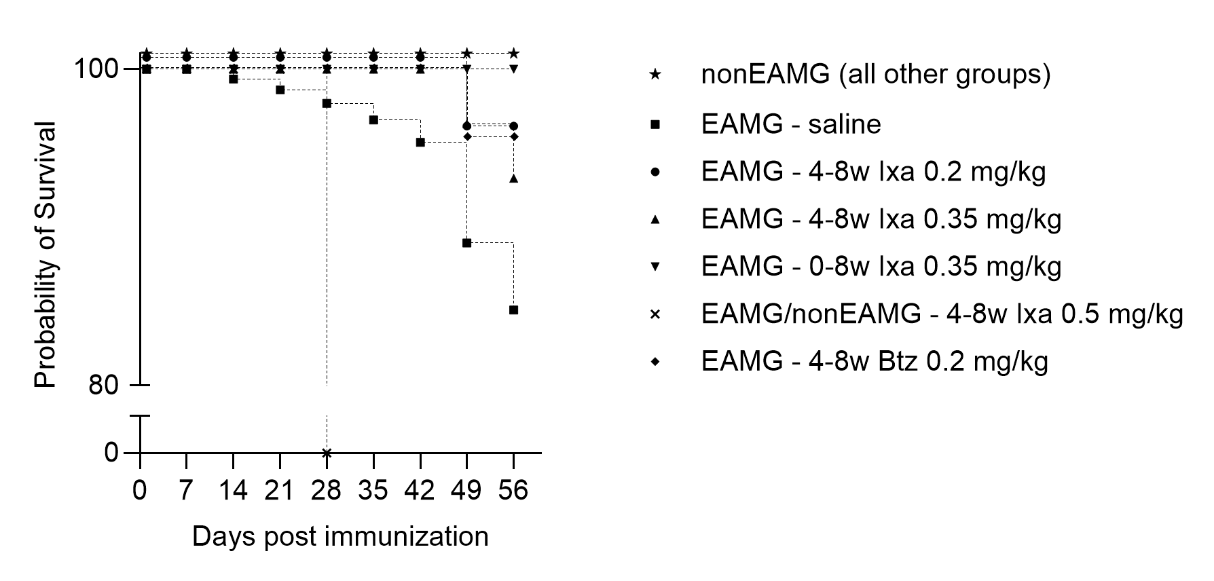


**Figure 2.** Kaplan-Meier Survival analysis. Data have been combined to include all animals in the study in the indicated groups. As expected, EAMG saline-treated animals had significantly more drop-outs than non-EAMG animals at either dose of ixazomib [p<0.0001]. After Ixazomib 0-8w Ixa 0.35 mg/kg, survival was significantly higher than in the EAMG saline-treated rats [p=0.0103]. Survival curves were compared using Long-rank (Mantel-Cox) test, Bonferroni post hoc testing.


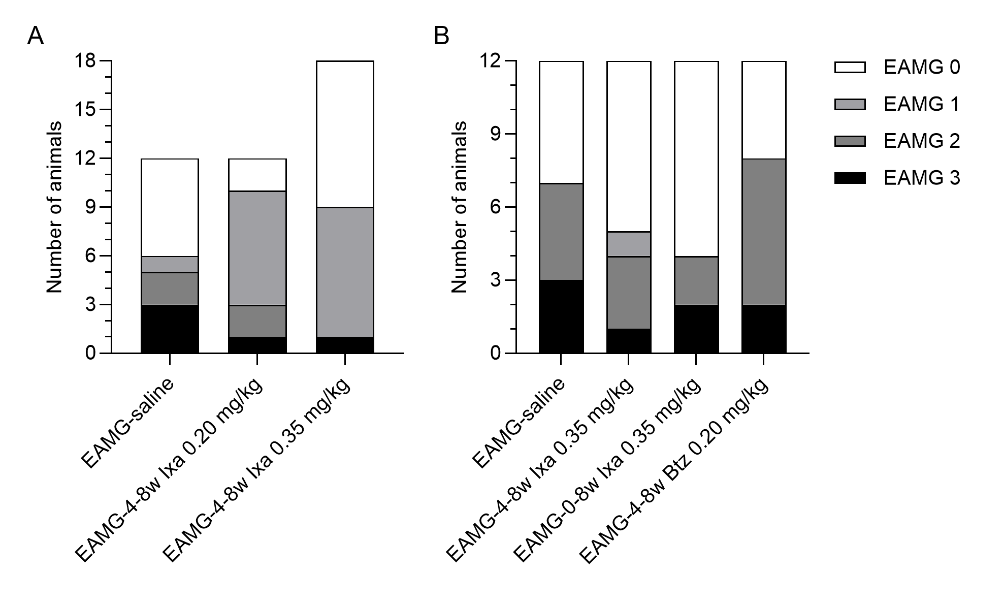


**Figure 3.** Clinical scores of muscle weakness at the end of the experiment [after 8 weeks or earlier if animals reached the humane endpoint] assessed by a blinded investigator. 0 = no abnormalities, 1 = fatigable weakness, 2 = constant weakness, 3 = severe muscle weakness or more than 20% weight loss. **A)** Dose-finding study and **B)** Treatment-timing study. Scores are indicated in percentages. No statistical differences were found on Kruskal Wallis testing.

**
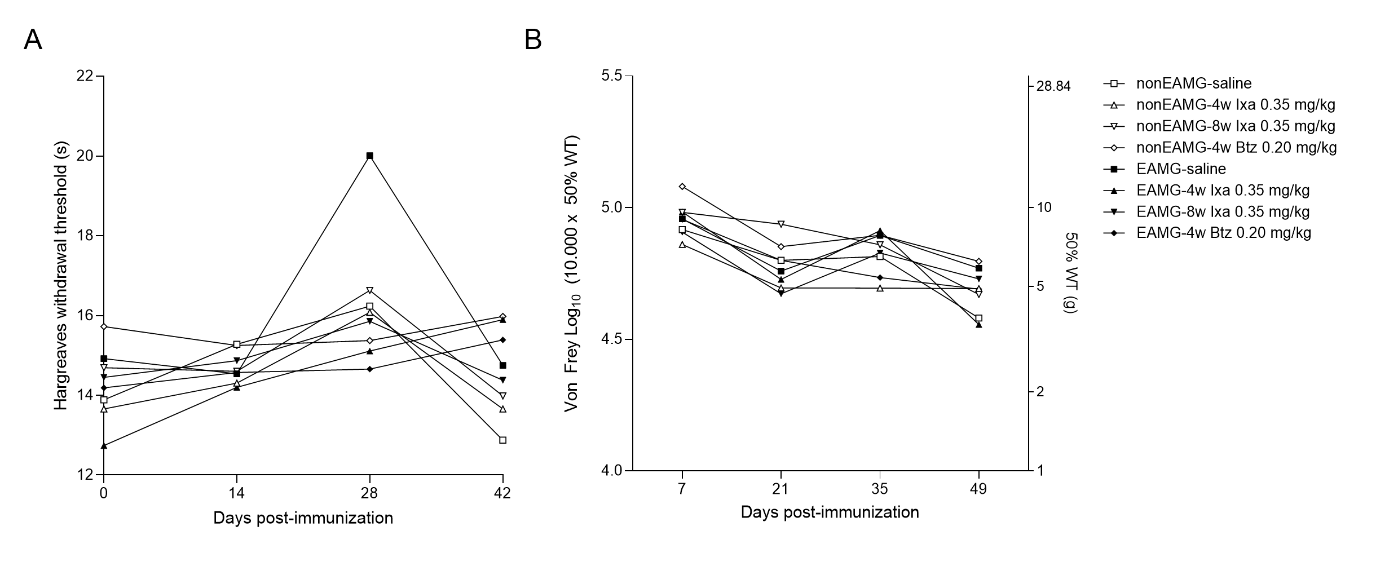
**

**Figure 4.** Pain-sensitivity tests. **A)** Hargreaves and **B)** von Frey tests performed on alternate weeks during efficacy study. Results show means.


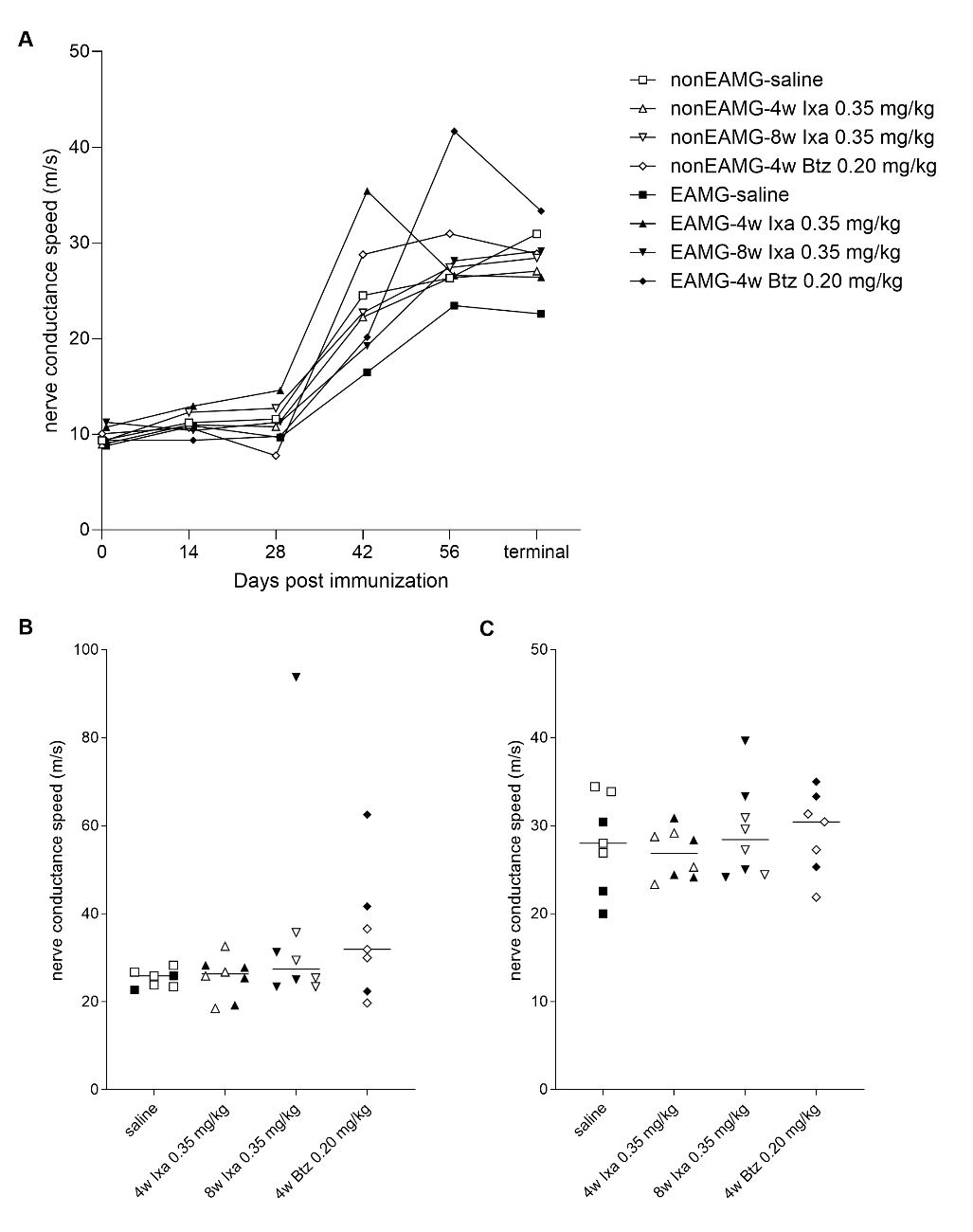


**Figure 5.** Nerve conductance speed measured in the tail. **A)** Nerve conductance speed measured every other week during the timing efficacy study, **B)** representation of treatment groups 8 weeks after immunization and **C)** before euthanasia. Individual values and median.
